# Challenges for the care delivery for critically ill COVID-19 patients in developing countries: the Brazilian perspective

**DOI:** 10.1186/s13054-020-03278-7

**Published:** 2020-09-30

**Authors:** Jorge I. F. Salluh, Thiago Lisboa, Fernando A. Bozza

**Affiliations:** 1 Department of Critical Care and Postgraduate Program in Translational Medicine, D’Or Institute for Research and Education (IDOR), Rua Diniz Cordeiro, 30 – 3º andar, Rio de Janeiro, 22281-100 Brazil; 2 Programa de Pós-Graduação em Clínica Médica, Universidade Federal do Rio de Janeiro, Rio de Janeiro, Brazil; 3 Critical Care Department and Programa de Pós-Graduação em Ciencias Pneumologicas, Hospital de Clinicas de Porto Alegre, Universidade Federal do Rio Grande do Sul, Porto Alegre, Brazil; 4 Instituto de Pesquisa Hospital do Coração - HCor, São Paulo, Brazil; 5 Critical Care Lab, National Institute of Infectious Disease Evandro Chagas, Oswaldo Cruz Foundation, Rio de Janeiro, Brazil

## Background

The delivery of critical care is a major challenge for developing countries [1]. The inequity of access to an ICU bed, heterogeneous triage policies, a low staff/patient ratio and suboptimal adherence to evidence-based practices contribute to disproportionally high mortality of sepsis and acute respiratory distress syndrome in these countries [2–5]. In addition, limited step-down and specialized ward beds’ availability further widens the gap between critical and non-critical care inside hospitals.

As the COVID-19 pandemic spreads through the world, developing countries are challenged with the surge of pneumonia cases where up to 30% of all hospitalized cases will require ICU admission [6]. In August 2020, Brazil is a hotspot of COVID-19 with more than 100,000 deaths. Other Latin American countries such as Mexico, Peru, Colombia, and Chile are also among the 10 countries with most cases worldwide. Several factors seem to have contributed to the dramatic progress of the epidemic in the country. Initial measures of social distancing were adopted at the beginning of the epidemic in several states. However, the lack of central coordination and, at a certain point, the denial of the pandemic by a populist government meant that more effective measures such as lockdown were not adopted whereas use of unproven therapies such as hydroxychloroquine was encouraged. Also, the low availability of tests and progression towards the interior and peripheries of large cities made the epidemic hard to control causing overwhelming hospitals and ICUs.

## What are the challenges?

Despite its high absolute number of ICU beds [7], even in comparison with western European countries, the heterogeneous regional distribution and payor-based access are major barriers for a more equitable delivery of critical care. Although increases in the number of ICU beds were recently made across the country as preparation for the pandemic, they are still insufficient to compensate regional differences (the North region has 50% fewer ICU beds per capita as compared with the Southeast) or the imbalances between the public and private sector. The number of public ICU beds per capita is 72% lower than private sector ones, and only 22% of the population has access to private healthcare.

Additionally, ICU staffing is an important shortcoming in the COVID-19 pandemic in developing countries. First, the ICU staffing can be considered low compared to developed countries, the current national norm defines a minimum of 1 nurse for each 10 ICU beds, and nursing technicians are the central workforce in several ICUs. Add the fact of the significant turnover due to a large number of healthcare professionals who are sick due to COVID-19, and the need for healthcare personnel with ICU training increases with the new ICU beds implemented for the pandemic. Other aspect to take in consideration is also the increasing complexity due to changes in case-mix of ICUs, where a large number of patients with multi-organ failure surge simultaneously. Data from the Brazilian ICU registry (www.utisbrasileiras.com/en that covers 1/3 of all ICU beds in the country) shows in 2019 an average of mechanical ventilation rates around 19% and an increase to 41% with COVID-19 [8]. This sudden shift in case-mix and increases in severity of illness of ICU patients could partially explain the high mortality rates for mechanically ventilated patients with COVID-19. The recently published results of the registry, in 13,941 COVID-19 patients requiring ICU admission, show a mortality rate of 32% for all patients and 67% for those requiring MV. This high mortality represents an excess even if compared to the more recent data on sepsis epidemiology, where rates were approximately 55% [4]. Recently, clinical characteristics and outcomes of COVID-19 patients from National Registries of critical care in LMICs (Brazil, Argentina, Sri Lanka, India) and HICs (Australia, New Zealand, Netherlands) were made publicly available by an international benchmarking initiative (icubenchmarking.com). Overall ICU mortality rates are comparable (ranging from 26 to 33%, except for Australia/new Zealand with rates of 7.8%). However, when the mortality of ventilated patients is analyzed, it tends to be higher in LMICs.

**Fig. 1:**
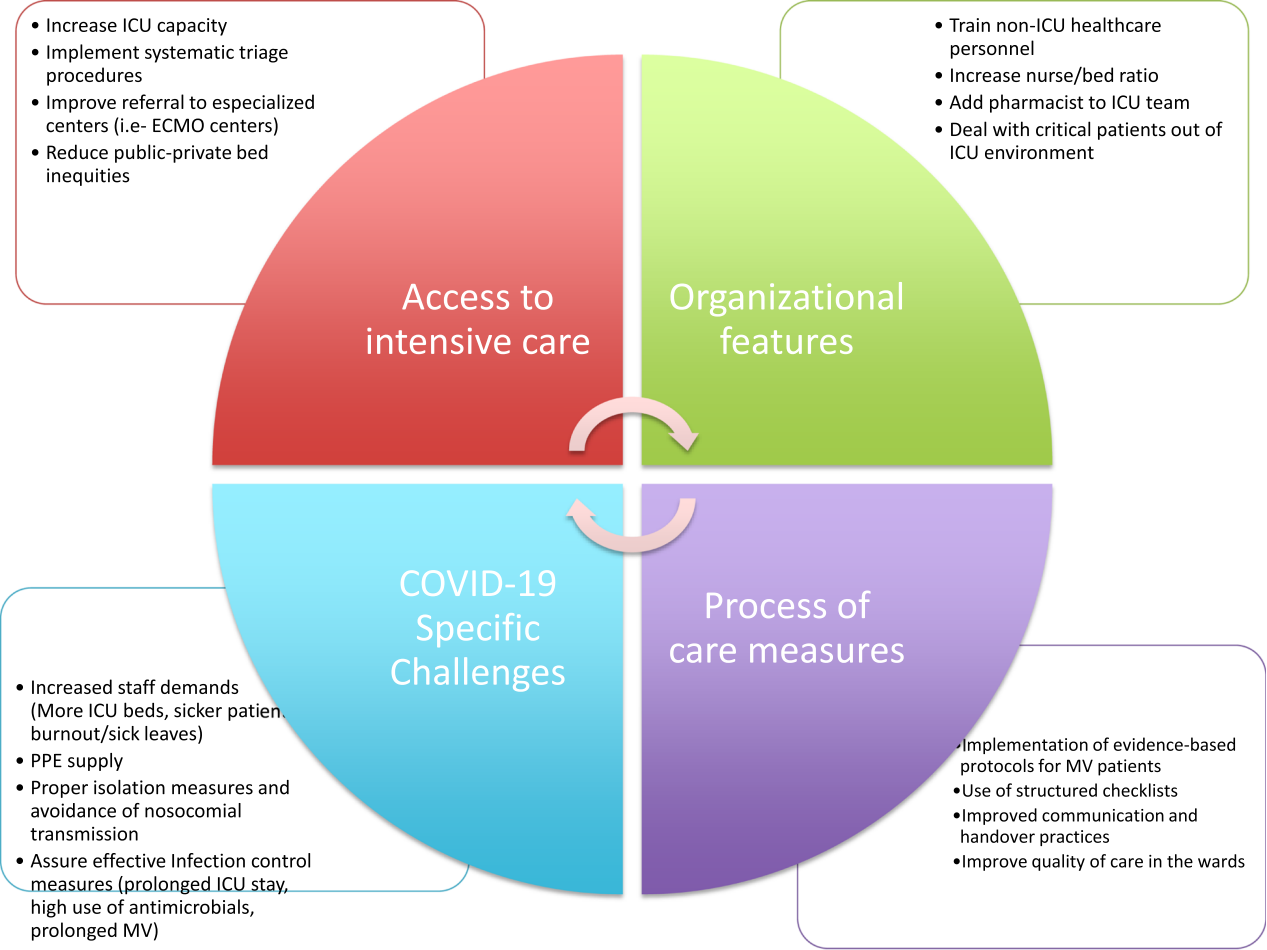
Challenges for the care delivery for critically ill COVID-19 patients in developing countries: the Brazilian perspective

In Brazil, lessons learned from the pre-COVID-19 period may be helpful and represent actionable information that hopefully can be translated into improved outcomes. Several studies performed in Brazilian ICUs demonstrate that there are opportunities to improve the quality of care. If the low baseline adherence to protective ventilation in ARDS [9, 10], sepsis protocols (less than 60% of patients) [5], or light sedation (less than 40% of patients) [9] may be seen as bad news, they also represent potentially modifiable factors (Fig. 1). Recent studies demonstrate that the use of quality improvement (QI) interventions is associated with improved outcomes in LMICs. Brazilian data shows that interventions such as early sepsis triage and treatment [5], the use of protocols for the prevention of ICU-acquired complications, and organizational changes (i.e., promoting autonomy for ICU nurses or adding a pharmacist to the staff) [11, 12] are associated with lower hospital mortality and lengths of stay. In addition, the use of a structured checklist during multidisciplinary rounds and the presence of an intensivist may improve adherence to sedation and protective ventilation protocols, both key factors to decrease the duration of ICU stay and to improve survival [13, 14].

## Conclusion

In conclusion, there are no easy solutions and developing countries such as Brazil need to fix the system from “access to bedside care” in order to improve outcomes during and after the pandemic. In this regard, better triage procedures and improved ward care could help to get the right patients to the ICU on time. Also, providing better ward care could minimize long-term complications due to physical and cognitive impairment in this population. Using proven quality improvement interventions should be #1 priority in the ICU as they represent a cost-effective strategy that usually does not require fancy technology or expensive drugs.

## Data Availability

N/A
